# Implementation and integration of image processing blocks in a real-time bottle classification system

**DOI:** 10.1038/s41598-022-08777-x

**Published:** 2022-03-22

**Authors:** Marco Aurelio Nuño-Maganda, Josué Helí Jiménez-Arteaga, Jose Hugo Barron-Zambrano, Yahir Hernández-Mier, Juan Carlos Elizondo-Leal, Alan Díaz-Manríquez, Cesar Torres-Huitzil, Said Polanco-Martagón

**Affiliations:** 1 Intelligent Systems Department, Polytechnic University of Victoria, 87138 Ciudad Victoria, Tamaulipas Mexico; 2 División de Ciencias Exactas, Instituto de Estudios Superiores de Tamaulipas, 89605 Altamira, Tamaulipas Mexico; 3 Facultad de Ingeniería y Ciencias, Universidad Autónoma de Tamaulipas, 87149 Ciudad Victoria, Mexico; 4 Tecnologico de Monterrey, School of Engineering and Sciences, Campus Puebla, 72453 Puebla, Mexico

**Keywords:** Engineering, Electrical and electronic engineering

## Abstract

A practical solution to the problems caused by the water, air, and soil pollution produced by the large volumes of waste is recycling. Plastic and glass bottle recycling is a practical solution but sometimes unfeasible in underdeveloped countries. In this paper, we propose a high-performance real-time hardware architecture for bottle classification, that process input image bottles to generate a bottle color as output. The proposed architecture was implemented on a Spartan-6 Field Programmable Gate Array, using a Hardware Description Language. The proposed system was tested for several input resolutions up to 1080 p, but it is flexible enough to support input video resolutions up to 8 K. There is no evidence of a high-performance bottle classification system in the state-of-the-art. The main contribution of this paper is the implementation and integration of a set of dedicated image processing blocks in a high-performance real-time bottle classification system. These hardware modules were integrated into a compact and tunable architecture, and was tested in a simulated environment. Concerning the image processing algorithm implemented in the FPGA, the maximum processing rate is 60 frames per second. In practice, the maximum number of bottles that can be processed would be limited by the mechanical aspects of the bottle transportation system.

## Introduction

There are many problems caused by water, air, and soil pollution, mainly produced by the large volumes of waste that receive inappropriate treatment. Recycling is a practical solution but sometimes unfeasible in underdeveloped countries, due to the lack of technical knowledge or economic resources required to carry out this task^1^. In recent years, plastic and glass bottles waste recycling is a critical issue, even when recycling is a relatively simple process. A typical bottle recycling process begins with cleaning and removal of contaminants. Next, it is required a manual separation by color. In the recycling field, a manual classification implies many security and health risks for the people performing the waste separation^2^.

Computer vision systems have three levels: the lower level includes the image capture and simple pixel-based functions such as arithmetical operations between images; the intermediate level includes more complex functions such as segmentation, matching, preprocessing, convolution, or motion estimation; the upper level typically is applied to recognize, classify or capture scene interpretation. Currently, there are a lot of FPGA-based computer vision systems that mainly employ parallel computing to increase the processing speed in these computationally intensive applications^3^. There are several implementations of color waste separation systems, but most of them have not been tested in a real environment with high accuracy and performance. A dedicated hardware solution has not being explored for solving this specific problem.

Commercial automated color sorting systems are expensive because the companies sell the vision system, the conveyor belts, and mechanical actuators in a single package. Usually, an automated system requires an industrial computer or a processing unit to classify bottles in one single line, because of the real-time limitations of sensors (cameras) and actuators to perform the object separation. The aforementioned implies more space and high power consumption, so the performance of the computer running the classification systems is not constant because of the lack of control in the number of concurrent processes. Although modern high-performance computer systems, including GPU devices, can be used to accurately classify glass or plastic bottles, these devices are not commonly used in recycling plants because both GPU and host computer require a lot of power.

## Related work

Many waste separator systems found in the literature are mainly focused on the mechanics of their systems. Other works are focused on the design of machine learning or computer vision systems. In this paper, those works based on the mechanical design are not reviewed.

An early bottle classification system did not use color information, but only the shape of each bottle using the height information using a laser range finder^4^. . They also developed an algorithm to detect stickers, which provides additional information to a classification system. Their approach was successfully applied when bottles have the same size, and slightly different shape. Other work proposes a mechanical system for separating cans, glass, and plastic bottles, aided by a computer vision system that sorts bottles by color^5^. The system classifies the cans into steel and aluminum and the plastic bottles by color and material. The authors do not provide details about the image processing algorithm.

Alternative approaches are separation systems consisting of magnet separation, particle size classification, vibration wind classification, bottle color separators, and plastic bottle separators^6^. The reported work focuses on the description of the separation of bottles and plastic. The authors reported a reflex-type discriminating system to handle non-opaque bottles frequently used for non-beverage products. A machine vision-based automated sorting was proposed^7^. They propose an image preprocessing stage, followed by feature extraction, bounding box computation, segmentation, and linear discriminant analysis (LDA). A dataset with 300 plastic bottle images is utilized, divided into two groups (PET and Non-PET).

The combination of near-infrared spectroscopy and color images in separated classifiers was done^8^. The first classification stage is the plastic-type classification using Near-infrared spectroscopy, while the second stage is color classification using machine vision techniques fed by a CCD camera. The plastic bottle type uses the power of a NIR signal, and a fusion of both quadratic discriminant function-based classifier and decision tree classifier to achieve the color recognition of the plastic bottles. The design and integration of the mechanical elements for automated sorting were addressed, but information about its physical testing with real-world images is missing^9^. Systems dedicated to identifying and extracting plastic bottles out of a conveyor belt using machine vision were explored^10^. The recognition system was tested on a set of 50 different bottles, obtaining an accuracy of about 97% on bottle identification, but the authors do not mention aspects related to the execution time.

Classifying recycled polycoat and PET materials was addressed by implementing a system based on the processing of gray images^11^. Image histograms are combined with an SVM classifier to segregate the input images. The authors report a high accuracy in the classification task, but, the complexity of the proposed classifiers makes their system unsuitable for real-time classification tasks. A system for sorting plastic and paper waste was proposed^12^. The proposed system processes high-resolution images later resized to a smaller resolution for its management. The system performs simple thresholding to separate objects from the background and a Feed-Forward (FF) Neural Network classifier to separate the waste. The thermoplastic-based recycling technique was explored^13^. The proposed system utilizes inductive, computer vision in the visible spectrum, and a NIR hyperspectral sensor to acquire the images required by a system that classifies thermoplastic using Artificial Neural Networks and PCA techniques. An Intelligent Waste Separator (IWS) to sort inorganic waste like plastic bottles, aluminum cans, plastic cutlery, and other kinds of materials was proposed^14^. The system classifies the target based on the first two Hu’s Invariant Moments (HIM) in conjunction with the k-Nearest Neighbor (k-NN) algorithm using euclidean distance. A system to classify a given plastic bottle image as PET or non-PET type was proposed^15^. Five feature extraction methods were implemented, including Principal Component Analysis (PCA), Kernel PCA (KPCA), Fisher’s Linear Discriminant Analysis (FLDA), Singular Value Decomposition (SVD), and Laplacian Eigenmaps (LEMAP). A Support Vector Machine (SVM) classifier performs the classification and a majority voting technique performs the final decision mechanism. The exploration of a Convolutional Neural Network (CNN) architecture named RecycleNet for classification of selected recyclable object classes was done^16^. The proposed approach reduces the parameters required in conventional CNNs architectures, but its drawback is the amount of data required for training, even with the optimized version proposed by the authors.

Many related works focus on the classification of multiple types of materials, requiring complex classifiers to achieve this task. On the other hand, few works focus their efforts on classifying only bottles (plastic or glass). Most of these works process low-resolution images. About the learning techniques, most works use complex learning techniques, whose main drawback is parameter tuning to reach high accuracies. One common choice is techniques based on deep learning which requires training using a large image dataset. Other works proposed systems that require small image datasets for training, but the accuracy is low. Most works perform offline processing, which generates higher accuracies, but information about the processing speed. In this work, we propose a compact and tunable solution based on reconfigurable hardware, as an alternative to microcomputers or PC based systems, that avoids the performance issues and high power consumption of the latter. The proposed system does not require a training stage, since we designed a hardware architecture with minimal resources to perform the high-performance bottle sorting. The classifier is very simple since it is based on a default threshold set or new thresholds that a system administrator inputs using a remote interface. The previous preprocessing stage to analyze the input image and isolate the bottle pixels is implemented on dedicated hardware to guarantee real-time performance. The classifier is the last step in the processes pipeline. The proposed system analyzes the bottle image, and the classifier obtains the bottle color based on the predominant color in the bottle pixels using the provided image thresholds. The rotation or flipping of input bottle images does not affect the processing, analysis, or classification stages. Augmentation is not required since there is not a formal training phase. Data diversity is desirable but not necessary for the proposed system. The system uses a controlled illumination, so the default or user-defined thresholds do not change too much and, the systems perform the bottle classification task with acceptable accuracy levels.

## Materials and methods

### Motivation of using FPGAs

The main advantage of using an FPGA is that the system guarantees a real-time processing of each frame of the input, while other systems implemented in Single Board Computers (SBC) or microcomputers, can not guarantee this performance. The FPGA development kit used in this work has a daughter board that acquires video from the camera, while the FPGA performs the decoding and processing without requiring external peripherals. We choose an FPGA for the proposed system because it allows us to implement custom hardware blocks for the image processing operators required in this project. The small disadvantage is that FPGAs are harder to program, compared to microcontrollers or microcomputers, since the programmer requires domain of digital hardware and microprocessor design concepts not required in high level languages. Related to power consumption, it is true that embedded computers (such as microcontrollers or SBC) consume less power compared to FPGA devices, however they do not provide the performance of dedicated hardware elements offered by FPGA devices. In the literature, a performance increase is always reported when using FPGAs for the implementation of image or video processing, compared to equivalent systems implemented in embedded computers. In the other hand, it is possible to use a PC with a powerful GPU to boost the performance of a system, but the power consumption will considerably increase, and the size of these systems is bigger and unsuitable for embedded applications.

### Hardware and software components

The validation of the proposed bottle color classification system was carried out on a Spartan-6 Industrial Video Processing Kit (S6IVPK), which hosts a Spartan-6 LX150T FPGA device. The S6IVPK is a targeted design platform. It includes two daughter boards based on the industry-standard FPGA Mezzanine Card (FCM) specification. The first one is the FMC-DVI, which allows the video processing generated by any video or still camera with HDMI output, and displays the resulting video in any video monitor with a DVI or HDMI input port. The second board is the FMC-IMAGEOV, which allows the video processing generated by an Omnivision OV7715 image sensor, and displays the resulting video in any video monitor with a DVI or HDMI input port.

As a capture device, a high-resolution digital camcorder connected to the FMC-DVI card was used. The HDMI output resolution was set to an image format of $1920 \times 1080$ pixels at 60 frames per second, also known as *1080 p*. An LCD monitor was connected to the FMC-DVI output port. From the S6IVPK, the RS-232 port with a DB9 connector receives the signal from the presence detector. The other RS-232 port was connected, through a USB converter bridge, to a computer to receive the configurable parameter using a 9600 bps serial protocol, and a data width of 8 bits. On the side of the host computer, a *Python* script sends the data with the proper format and configuration. The camera captures the bottle images from a stage with controlled illumination and white background (simulating a chamber), where several types of bottles were placed to determine their color. In the preliminary tests, the bottles were placed manually in front of the camera, but currently a fruit grading conveyor is being modified to accept bottles and install the proposed classification system.

A Laptop PC was used to configure, design, implement and debug the hardware blocks of the FPGA of the system. This Laptop requires the Xilinx ISE design suite installed. A Python script was programmed to send the configuration parameters to the FPGA device and to retrieve the output image processed by the FPGA, for debugging purposes.

### Required computer vision operations

For the implementation of the proposed bottle classification system, the following image processing operations were implemented.

- *Morphological processing*. A morphological process extracts the shape or structure of the objects in an image.
- *Spatial filtering*. The main objective of image enhancement is to process an image so, the result is more convenient than the original image for a specific application.
- *Color space conversion*. A color space is a method used to specify, create and visualize color. Brightness, tone, and color intensity features define a color. Usually, a color is specified using three coordinates or parameters. These parameters describe the location of the color in the chosen color space. he HSL color space represents a wealth of similar color spaces, whose alternative names include HSI (intensity), HSV (value), HCI (chroma/colorfulness), HVC, TSD (hue, saturation, and darkness), etc. Most of these color spaces are linear transforms from RGB and are therefore device-dependent and nonlinear. Their advantage lies in the extremely intuitive manner of specifying color.

### Proposed hardware blocks

**Figure 1:**
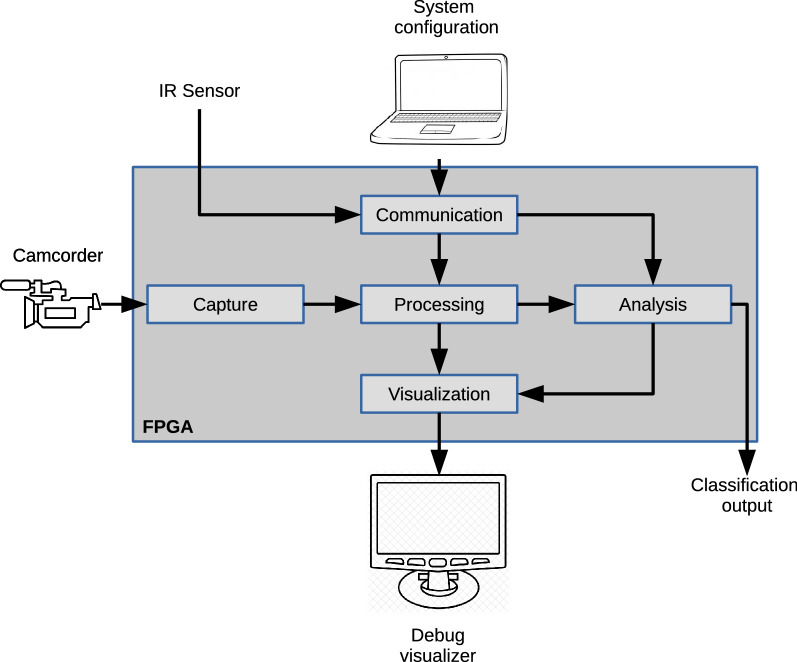
Block diagram of the proposed architecture.

Following the general schema of a typical computer vision system, a structure composed of several concurrent blocks working simultaneously was adopted to take advantage of the benefits offered by a custom hardware-implemented system. The architecture contains processing modules organized into groups depending on their function. Figure 1 shows the block diagram of the complete architecture.

The *capture block* is the main input interface, where the video signal is received. This module captures a video stream from an HDMI source and configures a predefined capture module using the I2C protocol. This design was adapted from^17^, using a provided reference design by the target board manufacturer and does not alter the video data, but only makes a passthrough function between the input and output HDMI ports. It provides a bus that carries the data signal in a 24-bit RGB format and the synchronization signals obtained by the camcorder.

**Figure 2:**
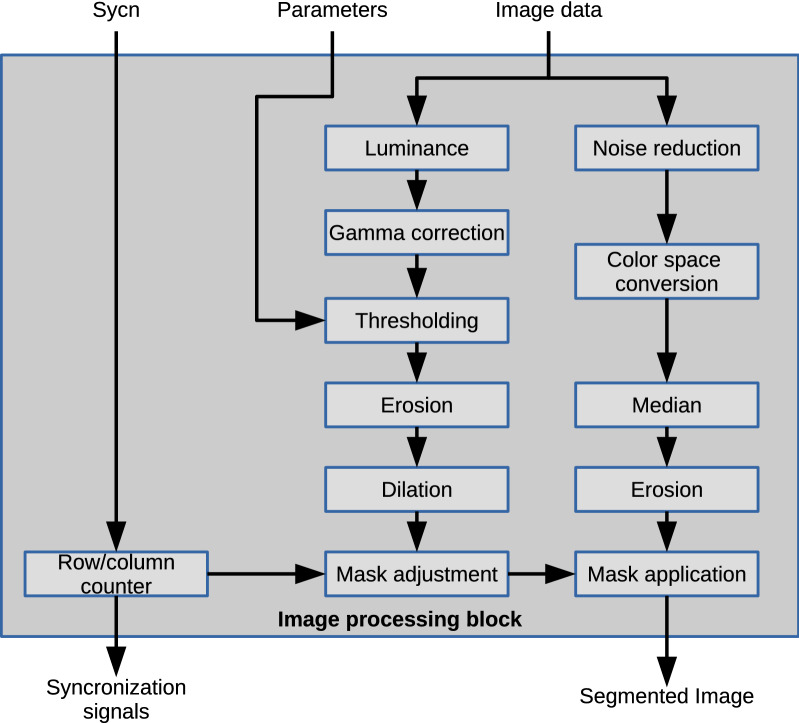
Block diagram of the image processing module.

The *processing block* performs all the digital image processing tasks. The processing block consists of modules that perform image processing tasks. The capture device generates the image without performing any modifications. This stage aims to process the image to facilitate further analysis. The suitable operators for the desired tasks were selected based on off-line tests. Figure 2 shows the block diagram of the image processing stage.

The *communication block* is an interface that receives data from a computer to configure parameters in the processing block and receives the signal from an external presence detector. The *analysis block* decides the membership group of the bottles being captured based on the data generated by the *processing block*. Finally, the *display block* generates the video signals displayed on an external monitor. In the final installation of the proposed bottle classification system both, system configuration and debug visualizer components, can be removed from the system.

Three main hardware blocks work in parallel in the processing stage. The first one receives the original image data and perform a noise reduction operation by using the intensity slice technique for each color channel.

The next module performs a color space conversion from the RGB to HSL color space. The module works only with the Hue channel, in order to process less information.

Another noise reduction filter used was the median filter, which uses a convolution scheme. To simplify the circuit, a modified version of the noise reduction filter is used. The original algorithm sorts all the pixels within the processing window in a decremental or incremental way, and then, the median is selected. The proposed method groups the pixels within the window into smaller groups, and each subgroup passes through comparisons blocks to obtain the median from each group. The resulting medians feed another comparison block that gives the median value as the output for this process.

The morphological filters were designed in order to be flexible concerning the data width, so the filter adaption to binary or grayscale images is straightforward. The dilation and erosion filters work similarly to convolution, using a processing window. In the proposed median filter implementation, the dilation uses a comparison block that receives all the inputs from the processing mask. The comparison returns the maximum value as the dilation output. The erosion block returns the minimum value, having the same organization as the dilation block.

To prepare a mask for segmentation, the first step in this processing branch is the conversion from RGB components to the luminance component of the HSL color space.

This conversion generates a grayscale image. The dedicated processing module receives the 24-bit data signal from the RGB channels, 8-bit each. A control block obtains the maximum and minimum values and performs a right shift to finally add the values and generate an 8-bit result for the luminance channel representation.

This module uses the corrected image to generate a binary mask to ignore all those pixels out of the region of interest. After obtaining the last grayscale image, the module segments this image using a constant thresholding level. The mask image determines object regions using a 1-value for those pixels that belong to the object and a 0-value otherwise. This module also applies the resulting mask to the last image from the first processing step.

The third stage of the processing module receives the synchronization signals from the external capture device. Two binary counters (column and row counters) use the horizontal and vertical synchronization pulses as clock signals. These counters compute the display coordinates of the current pixel. In this block, the video data is not affected. The information about columns and rows limits the object area in the image mask.

**Figure 3:**
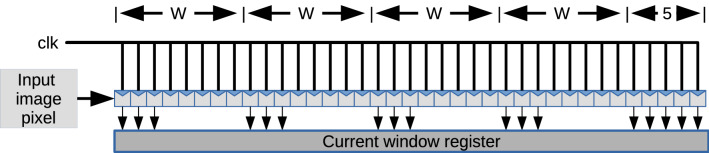
Block diagram of the shift register used for image enhancing.

An image window performs filtering operations. In the proposed implementation, a shift register that stores four rows of the input image to have full access to the first five rows of the input image is used. The latter is required to keep the input image in external memory, so the length of the registers depends on the target resolution. Figure 3 shows the architecture of the proposed shift register. The output of this shift register feeds the mean filtering block in the processing block.

**Figure 4:**
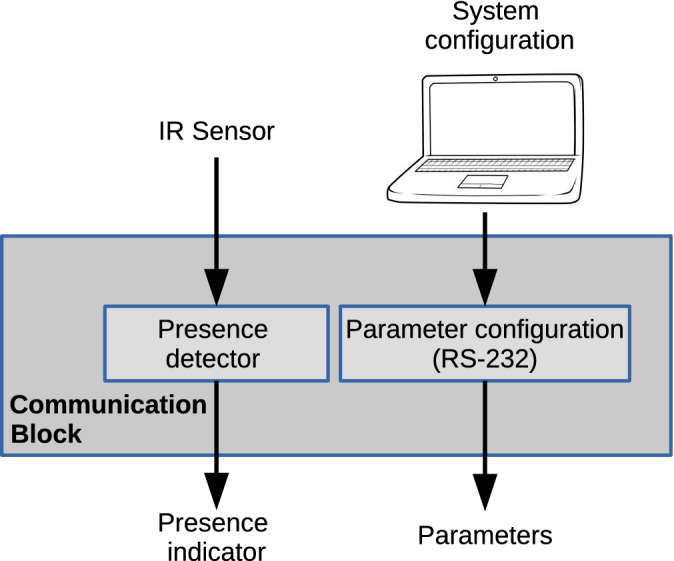
Block diagram of the communication module.

A communication block to establish communication between the FPGA board and an external device was designed. This block is composed of two functions. The first one provides a source signal to a presence detector and receives the response signal indicating if there is a bottle or not. The second one establishes serial communication with a computer using the RS-232 protocol to receive parameters information. To interface the proposed architecture with an external bottle detection circuit, one input/output port available in the board to connect the FPGA is used. This circuit was built using an infrared sensor with a phototransistor and an IR LED. The sensor delivers a logic signal when an object obstructs the transmitter–receiver vision line. Internally, this presence signal is used in the analysis stage to determine when a bottle is present. The USB-UART bridge available on the FPGA development kit was used to implement an RS-232 protocol with a finite state machine (FSM). It receives 8-bit data to configure the threshold value as a variable parameter. The FSM has nine states, one is used for the idle state, including the start and stops bits and saves the needed number of states. The other eight states are used to store each bit of the transmitted data. When the FSM reaches the idle state, the module sends the stored byte to the processing block to configure the corresponding parameter. The FSM works under its clock domain at a speed of 9.6 kHz for a data transmission rate of 9600 bps. The FPGA generates this clock from the FPGA clock signal that oscillates at 100 MHz. Figure 4 shows a block diagram including the presence sensor and the serial communication block.

**Figure 5:**
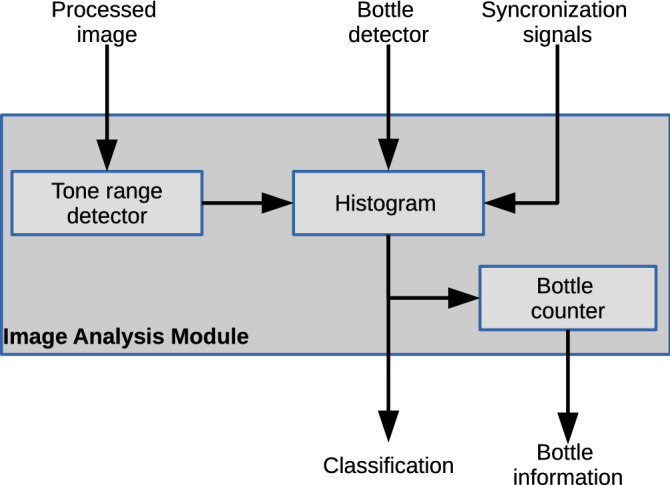
Block diagram of the analysis module.

This analysis block receives the processed image to compute the classification of the object depending on its predominant color. Input data is analyzed to establish the color of each pixel. Later, this module generates a histogram to compare occurrences of each intensity level considered. When a bottle is detected, and once classified by color, a counter will increase its value. The counters keep a record of the total quantity of bottles of each color group. Figure 5 shows the block diagram for the analysis module. The processed image is compared in the tone range selector to assign a color group based on the input pixels. The four possible groups are: brown, green, blue, and transparent. Before the implementation of the proposed architecture, we determine the hue range for each color. This block uses this range to classify each pixel color. When the module cannot compute the predominant color, the transparent color is assigned. The histogram generator block reads each 4-bit register that stores the color counters to determine the final classification.

The histogram generator uses the signal generated by the presence detector to obtain the current pixel color. The module obtained the predominant color group once the module analyzed the current frame. One 4-bit register stores the bottle color classification, with a single bit of this register corresponding to each group to be classified. The next block uses the final classification register to count the number of bottles of each group. The bottle counter block has a binary counter with a programmable delay based on the bottle speed. This delay can be adjusted in execution time depending on the hardware capabilities of the conveyor band attached to the vision system.

**Figure 6:**
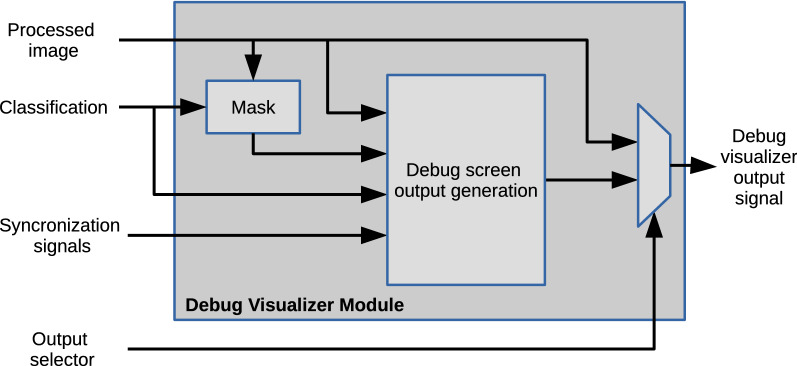
Block diagram of the display module.

The display module receives data from the processing block, the classification variable, synchronization signals, and it has a connection with an external DIP switch. This block performs a final mask adjustment and generates the data signals for on-screen information display. Figure 6 shows the block diagram for the display module.

The final mask adjustment receives the binary image and gives output data according to the color of the classification made for the previous frame. Internally, this is done through a multiplexer with constant inputs representing the classification colors in a 24-bit RGB format for each group. The classification register controls the output selector, and it is enabled when the signal from the mask has a value of 1.

The display module information block generates a graphical representation of the information obtained in the previous processes. It selects the color data sent to the output as a video signal. A set of ROM blocks (ROM32X1 elements) were instantiated with the required values to display the digits for counting the bottles. The system uses an array of $32 \times 16$ bits to show digital information, where a 0-value represents background pixels and a 1-value the foreground. The current bottle counter reads data from eight ROM instances (ROM32X1) that contain the $32 \times 8$ pixels icon. The color of the icon is selected depending on the classification of the bottle in the capture area. The system displays this icon in the same row as the corresponding bottle counter to identify the quantity of bottles of each group.

## Results and discussion

**Figure 7:**
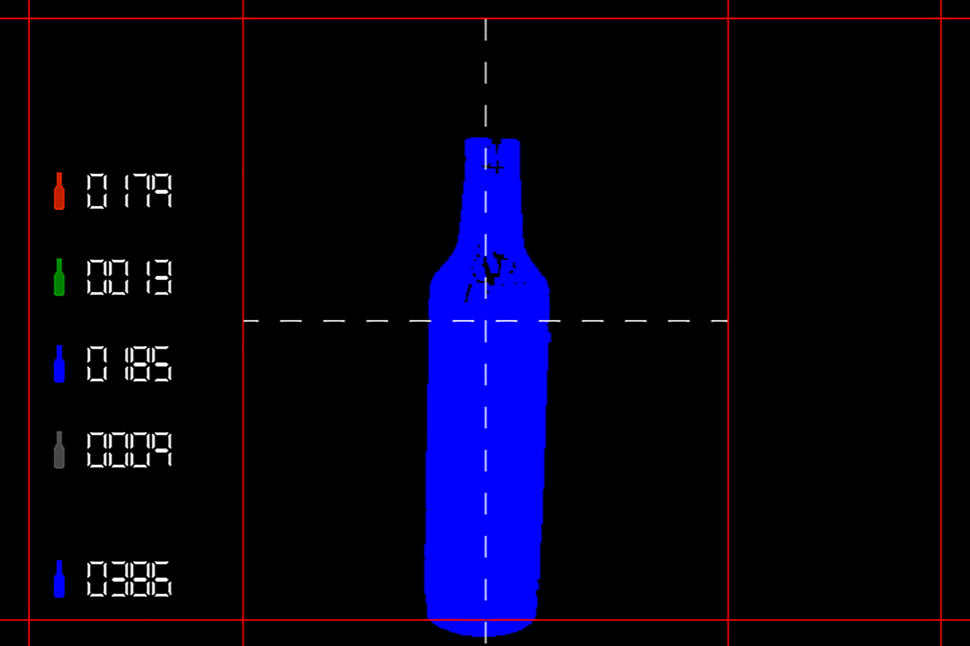
Final image displayed on screen.

When the target FPGA uses the default switch configuration, the displayed image shows the final classification result resulting from the processing and analysis tasks. The image contains a set of graphical elements to show the total quantity of processed bottles, as well as, the quantity by groups, including an indicator icon for each group and the current classification of the bottle being processed. The biggest image area displays the last color classification after the processing. Figure 7 shows the default image, and the results generated by all the previous modules. We considered four different bottle colors for testing because these colors were available. The proposed system is configurable to sort several bottle colors by using a software application that connects to the FPGA board using a serial connection. The system’s administrator loads an image set that contains the bottle color to be added, and the software solution analyses the image dataset to estimate the image thresholds. Finally, these thresholds are transferred to the FPGA board. For testing purposes, we tested with four different bottle colors.

The final report of the FPGA hardware design yields a low consumption of slice registers. The required LUTs, used mainly for logic and memory purposes, represent only 4% of the total available. The occupied slices represent an utilization of less than 7% of the total number of available slices. The IOBs are resources for input and output signals, mainly used in the capture module to connect to the input camera and the output monitor and to interface the communication module with the serial ports. The RAM16BWER is a memory resource used only for the capture module. The DSP48A1s are resources with adders and multipliers on its internal structure; two of these resources are used in the final implementation to perform the multiplication operations required by the color space conversion block.

Final experimentation test were made on the video resolutions shown in Table 1. For each resolution, the time to complete the bottle classification was estimated based on the pixel frequency. Even when the processing time is short, the performance of the system is limited by the video frame rate, which for all the tested resolutions, was around 16.7 ms. For comparison purposes, an equivalent processing pipeline was programmed in a PC, using the C++ language and the OpenCV library. When using a 480 p resolution, a 188 ms execution time was obtained, that translates in a processing rate of 5.319 frames per second. In the FPGA implementation, if a higher resolution input video is used, the processing speed of the whole frame remains constant. In opposition, in the software implementation, the processing time will proportionally increase with the input frame resolution. In the FPGA implementation, if a higher resolution input video is used, the processing speed of the whole frame remains constant. In opposition, in the software implementation, the processing time will proportionally increase with the input frame resolution. For each implemented video resolution, we estimate the used logic resources, which are summarized in Table 2.

**Table 1:** Execution time of the bottle classification process at several image resolutions.

Video resolution	Pixel freq (MHz)	Frame dimensions	Execution time on FPGA (ms)
480 p	25.125	$640 \times 480$	12.35
720 p	74.250	$1280 \times 720$	12.49
SXGA	110.000	$1280 \times 1024$	11.97
1080 p	150.000	$1920 \times 1080$	13.88
UXGA	165.000	$1600 \times 1200$	11.68

**Table 2:** Summary of results for different video resolutions.

Video resolution	Slice registers	Slice LUTs	Memory	Logic LUTs	Occupied slices	RAMB16-BWERs	DSP-48A1s
480 p	732 (1%)	4271 (4%)	2932 (3%)	1325 (6%)	1438 (6%)	1 (1%)	2 (1%)
720 p	730 (1%)	4982 (5%)	2688 (2%)	2278 (10%)	1683 (7%)	1 (1%)	2 (1%)
SXGA	730 (1%)	4977 (5%)	2686 (2%)	2278 (10%)	1661 (7%)	1 (1%)	2 (1%)
UXGA	730 (1%)	5821 (6%)	2964 (3%)	2837 (13%)	1974 (8%)	1 (1%)	2 (1%)
1080 p	3919 (2%)	7724 (8%)	4407 (4%)	3298 (15%)	2685 (11%)	1 (1%)	2 (1%)

### Comparison with related work

The main contribution of this paper is the implementation of a high performance image processing system for bottle classification. The image processing techniques presented in this article were chosen so they could be implemented in hardware modules and achieve real-time performance. Additionally, the proposed system does not require a training stage and the “model” is stored as a set of threshold values instead of a complex representation that cannot be updated by the final user. Moreover, it does not require to reduce image resolution before the image processing stage. These hardware modules were integrated into a compact architecture and tested in a simulated environment. The architecture allows the user to choose the bottle colors to be classified using a PC application that communicates with the FPGA through a serial port. The system was tested in a simulated scenario that emulates bottles on a conveyor.

To keep the hardware resources for the implementation of the proposed classification system as low as possible, a scheme to keep a low number of steps for the selected classification was adopted. Complex classification techniques based on supervised algorithms have been discarded because of the added complexity to the system in detriment of the performance. In the proposed approach, the image preprocessing tasks to enhance the input images were simplified to reduce the final hardware requirements.

To compare the proposed work with existing systems, we have summarized in Table 3 the remarkable points. Some works use complex learning techniques, such as NN^7,12,16,18^, LDA^9^, Decision Tree classifiers^8^, SVM^11,15^, KNN^14^ and SOM^10^, applied in different domains. The main problem with this techniques is the tuning of the parameters to reach useful accuracies. The image processing techniques used in this work were chosen so they could be tunable and reliably programmed in hardware, without sacrificing the overall performance. Some works use deep learning approaches (DL)^16,19^, which must be trained with a great number of images. Although, multiple resources are available on the internet, a customized DL solution needs the labeling of hundreds or perhaps thousands of images, which involves time and resources that are not available at the time of the system development. Moreover, the power requirements of these systems are high. On the other hand, the proposed approach is based on the selection of color thresholds that can be selected by the final user and does not require a training stage. Additionally, the power requirements are low, thanks to the use of an FPGA as processing unit. Some works proposed a minimal system using few training images^4,7,10,11,14,15,18^, but the resulting classifier accuracy is low. Some related systems do not provide information about the used classifiers or the images used to train those classifiers^5,8,12,20^. The proposed system does not require a training stage. It was tested in controlled conditions with bottles of four different colors and, in all cases the classification results were correct. Some works process images offline^4,7,9–12,15,16,18^, while the proposed work process video in real-time. This offline processing gives the proposed system an advantage since they report higher accuracies, but information about the processing speed does not guarantees a real-time performance with their current implementation. Some related works process video at small resolutions^5,6,20^, however they do not provide information about the video format or the type of interpolation applied. The proposed approach processes video in its native resolution, so interpolation techniques are not needed. Some of the related works process still images at low resolutions^7,12,15,16,18^. Furthermore, they trained their classifiers with these low-resolution images. They probably do not test their implementation at higher resolutions because the complexity of the required operations does not allow them to reach real-time performance. Nevertheless, our approach is able to process video at 60 fps at the full camera resolution. The proposed architecture can be reconfigured to separate several types of bottles based on the requirements of the waste separation authority, by only tuning some parameters, avoiding the need to retrain the whole system. The proposed system is based on a FPGA. FPGAs are useful in several domains that require the high-performance image processing and low power consumption. Related works found in the literature do not use FPGAs. We suppose that this is because the time and effort required to learn to program FPGAS are longer, compared to programming PCs or microcontrollers.

Due to the image processing operators, the proposed system is able to classify only non-deformable containers, such as rigid cans, or plastic and glass bottles, and it would not be possible to classify plastic bags, organic waste or compacted materials.

The proposed system is not able to process unsorted bottle groups or overlapped bottles. It can only process images of individual bottles. Hence the need to use a transport density in the conveyor of 25% to ensure the processing of one bottle per image.

The proposed system is more expensive than SBC and microcontroller based systems, but it is still affordable compared to other systems requiring PC with or without GPU, which are also more power demanding and bulkier.

## Conclusion and future work

A hardware architecture for the classification of bottle images for a recycling line was developed. The architecture contains several processing modules, which can be used as individual blocks or black boxes to perform specific tasks and be included in other designs.

The image processing tasks implemented on an FPGA are fast enough to be part of a real-time processing system. This hardware-based processing saves time because the storage of the complete image is not necessary. It also saves memory resources because the architecture only requires the storage of data needed for the window processing tasks, in this case, four lines and five pixels from the image. Compared to state-of-the art implementations on PC, the variations in the results produced by algorithm modifications for their implementation in FPGAs are minimal.

The hardware architecture designed is an efficient solution for object classification by color, and presents a high performance and enough features to be integrated into an industrial image processing system, not only for color sorting. The classification parameters can be easily adjusted using a laptop computer, and modifications to the target recognition colors for recognizing several types of targets are straightforward.

**Table 3:** Comparison of proposed system with systems reported in the literature.

Work	Classifier type	Platform	Material types	Coupled to separator machine	Dataset	Accuracy	Sensor type	Image resolution
^16^	CNN	PC and GPU	Six materials	NO	TrashNet	95%	RGB	224x224
^4^	Image Processing	NA	Plastic glass bottles	NO	66	100%	Laser range finder	N/A
^5^	N/A	N/A	Cans, Glass and plastic bottles	YES	N/A	N/A	RGB	N/A
^6^	N/A	N/A	Glass and plastic bottles	YES	NO	N/A	RGB and Spectroscope	N/A
^7^	NN	N/A	Plastic bottles	NO	100	96%	RGB	$256 \times 356$
^9^	LDA	N/A	PET and Non-Pet glass bottles	NO	300	97.5%	RGB	N/A
^8^	QDA and Tree classifiers	N/A	Plastic bottles	YES	N/A	83.48%	NIR and RGB	N/A
^10^	SOM and NN	PC	Plastic bottles	NO	50	97%	RGB	N/A
^15^	SVM	N/A	Plastic bottles	NO	90	90%	RGB	$320\times 240$
^14^	KNN	N/A	Plastic cutlery, bottlesand cans	YES	60	98.33%	RGB	$1280\times 1024$
^12^	NN	N/A	Plastic and paper materials	NO	N/A	84.44%	RGB	$100\times 140$
^11^	SVM	N/A	Polycoat containers and plastic bottles	NO	48	93.7–98.1%	RGB	$640\times 480$
^19^	R-CNN	PC & GPU	40 types of garbage	NO	3,984	93.3%	RGB	N/A
^20^	N/A	N/A	Plastic and glass bottles, Cans	YES	N/A	95%	RGB, NIR and Barcode	N/A
^18^	NN	N/A	Plastic, glass and paper	NO	100	95%	RGB	$400\times 200$
This work	N/A	N/A	Glass and plastic bottles	NO	N/A	N/A	RGB	$1920\times 1080$

The proposed hardware architecture was synthesized for several standard video resolutions to give the final user flexibility about the image sensors that could be used in the implementation of the system. The target architecture was tested for several video resolutions, but the architecture adaptation to process higher resolutions such as 4 K or 8 K is straightforward when this technology will be available to be included in some system.

As future work, the communication block functionality to configure several parameters could be modified to be able to transfer information from the FPGA about the current values for the different parameters and data resulting from the analysis tasks. Another improvement is to explore the FPGA internal clock to perform intermediate operations faster because the image processing module works under the video clock domain.

Additionally, from the state-of-the-art related work, we are going to explore the adaptation of the proposed system to a machine similar to that reported in^6^, where bottles travel lying down, since we have access to a fruit grading machinery that would require only sightly modifications to the conveyor to be able to transport bottles.
